# Lifetime excess absolute risk for lung cancer due to exposure to radon: results of the pooled uranium miners cohort study PUMA

**DOI:** 10.1007/s00411-023-01049-w

**Published:** 2024-01-03

**Authors:** M. Kreuzer, M. Sommer, V. Deffner, S. Bertke, P. A. Demers, K. Kelly-Reif, D. Laurier, E. Rage, D. B. Richardson, J. M. Samet, M. K. Schubauer-Berigan, L. Tomasek, C. Wiggins, L. B. Zablotska, N. Fenske

**Affiliations:** 1 Federal Office for Radiation Protection (BfS), Munich (Neuherberg), Germany; 2 National Institute for Occupational Safety and Health, Cincinnati, OH USA; 3 Occupational Cancer Research Centre, Toronto, Canada; 4 Institute for Radiological Protection and Nuclear Safety (IRSN), Fontenay-aux-Roses, France; 5 University of California, Irvine, CA USA; 6 Colorado School of Public Health, Aurora, CO USA; 7 International Agency for Research on Cancer, Lyon, France; 8 National Radiation Protection Institute, Prague, Czech Republic; 9 University of New Mexico, Albuquerque, NM USA; 10 New Mexico Tumor Registry, Albuquerque, NM USA; 11 University of California, San Francisco, CA USA

**Keywords:** Uranium miners, Cohort study, Lung cancer, Radon, Mortality, Lifetime risk

## Abstract

The Pooled Uranium Miners Analysis (PUMA) study is the largest uranium miners cohort with 119,709 miners, 4.3 million person-years at risk and 7754 lung cancer deaths. Excess relative rate (ERR) estimates for lung cancer mortality per unit of cumulative exposure to radon progeny in working level months (WLM) based on the PUMA study have been reported. The ERR/WLM was modified by attained age, time since exposure or age at exposure, and exposure rate. This pattern was found for the full PUMA cohort and the 1960 + sub-cohort, i.e., miners hired in 1960 or later with chronic low radon exposures and exposure rates. The aim of the present paper is to calculate the lifetime excess absolute risk (LEAR) of lung cancer mortality per WLM using the PUMA risk models, as well as risk models derived in previously published smaller uranium miner studies, some of which are included in PUMA. The same methods were applied for all risk models, i.e., relative risk projection up to <95 years of age, an exposure scenario of 2 WLM per year from age 18–64 years, and baseline mortality rates representing a mixed Euro-American-Asian population. Depending upon the choice of model, the estimated LEAR per WLM are 5.38 × 10^*−*4^ or 5.57 × 10^*−*4^ in the full PUMA cohort and 7.50 × 10^*−*4^ or 7.66 × 10^*−*4^ in the PUMA 1960 + sub-cohort, respectively. The LEAR per WLM estimates derived from risk models reported for previously published uranium miners studies range from 2.5 × 10^*−*4^ to 9.2 × 10^*−*4^. PUMA strengthens knowledge on the radon-related lung cancer LEAR, a useful way to translate models for policy purposes.

**Supplementary Information:**

The online version contains supplementary material available at 10.1007/s00411-023-01049-w.

## Introduction

Radon is an established lung carcinogen and an important occupational and environmental cause of lung cancer (UNSCEAR 2020). This was demonstrated in residential radon studies in the general population and in studies of uranium and other radon-exposed underground miners. Cohorts of uranium miners continue to form an important basis for radiation protection standards for radon progeny. They consistently show that the excess relative rate (ERR) of lung cancer mortality increases linearly with increasing cumulative exposure to radon progeny (in the following abbreviated to “radon”) in WLM and that the ERR/WLM is modified by attained age, time since exposure or age at exposure, and exposure rate (NRC 1999, UNSCEAR 2020). The calculation of the lifetime excess absolute risk (LEAR) allows the comparison of estimates of the ERR/WLM obtained from different studies with different characteristics while using the same exposure scenario and baseline mortality rates. Such estimates are also useful for policy considerations. The LEAR of lung cancer related to exposure to radon for example has been used in the past as the basis for the dose conversion convention for radon (ICRP 1993, 2010). This method has been used to convert measured radon activity concentrations into an effective dose in mSv, which is important to check the compliance with occupational radiation limits given in mSv. In this "epidemiological" approach of dose conversion, the LEAR of lung cancer per unit of exposure to radon progeny is divided by the detriment (representing the harm) per unit of effective dose (ICRP 2010). Determining the most appropriate value of this dose conversion coefficient has been the subject of much controversy in recent years (Harrison et al. 2020, 2021; Laurier et al. 2020; Marsh et al. 2021).

While previously an LEAR of 2.8 × 10^*−*4^ per WLM was assumed based on risk models derived from a meta-analysis of 7 miners cohort studies (ICRP 1993), this value was revised to 5 × 10^*−*4^ per WLM by the International Commission on Radiological Protection (ICRP) in 2010 (ICRP 2010) based on new risk models from a pooled analysis of 11 miners studies (BEIR VI study) (NRC 1999) and a pooled Czech/French study (Tomasek et al. 2008a). Both LEAR calculations used a mixed male/female Euro-American-Asian population for baseline rates of lung cancer mortality (ICRP 2007) and assumed an exposure scenario of 2 WLM per year between age 18 and 64 years. In 2020, the United Nations Scientific Committee on the Effects of Atomic Radiation (UNSCEAR) reviewed epidemiological studies and calculated the LEAR per WLM for death from lung cancer in a similar manner using data from four miners studies (UNSCEAR 2020; Tomasek 2020), among them for the first time the large German Wismut cohort (Kreuzer et al. 2018). The LEAR ranged from 2.4 (Wismut cohort) to 7.5 (Eldorado cohort) × 10^*−*4^ per WLM. Heterogeneity in radiation risk estimates between studies may explain differences in the LEAR and could be due to several factors: differences in the range of cumulative exposure and exposure rate, concomitant exposures to other lung cancer carcinogens, duration of follow-up and employment, methods of mortality follow-up, composition of the study population, existence and control for potential confounders, measurement error, loss to follow-up and competing risks for mortality, statistical power, and also statistical methods.

A major step forward was therefore the worldwide pooling of uranium miners studies, the Pooled Uranium Miners Analysis (PUMA) study (Rage et al. 2020; Richardson et al. 2021), which aims to get more precise estimates of the lung cancer risk associated with radon based on standardized statistical analyses of existing cohorts. PUMA includes twice as many uranium miners and about three times as many lung cancer deaths (Rage et al. 2020) as the pooled BEIR VI study (NRC 1999). The majority of included studies have an updated mortality follow-up and all studies follow a common study protocol and statistical methods. Recently, two papers on radon-lung cancer mortality associations among men in PUMA have been published, addressing: (1) the 1960 + sub-cohort of miners hired in 1960 or later (Richardson et al. 2022) with chronic low radon exposures and exposure rates mostly based on measurements, and (2) the full PUMA cohort (Kelly-Reif et al. 2023) including very high radon exposures from the early years of mining and low radon exposures in the later years.

The aim of the present paper is to calculate the LEAR per WLM for death from lung cancer using the new risk models based on the pooled data of the PUMA study and the risk models of previously published uranium miners studies, including the recently updated German Wismut cohort (Kreuzer et al. 2023), while using the same methods for all analyses. To be comparable to previous LEAR calculations as in UNSCEAR (2020) and ICRP (2010), the exposure scenario was defined as 2 WLM per year from age 18–64 years, and baseline mortality rates of the ICRP mixed Euro-American-Asian population (ICRP 2007) were chosen.

## Methods

### PUMA data

The PUMA study includes seven cohorts from Canada, the Czech Republic, France, Germany, and USA, which have been previously described in detail (Rage et al. 2020; Richardson et al. 2022; Kelly-Reif et al. 2023). The ERR/WLM was estimated in analyses of men included in PUMA based on the BEIR VI exposure–age–concentration model (NRC 1999, UNSCEAR 2020) and an alternative risk model (i.e., the BEIR VI model, but with age at exposure instead of time since exposure). The corresponding statistical methods and findings have been published for the full cohort (Kelly-Reif et al. 2023) and the PUMA 1960 + sub-cohort of miners hired in 1960 or later (Richardson et al. 2022). Main characteristics of both cohorts are described briefly in Table 1.

**Table 1:** Characteristics of the PUMA full cohort and PUMA 1960 + sub-cohort of miners hired in 1960 or later

	Period of follow-up	Number of miners	Number of lung cancer deaths	Mean duration of employment (years)	Mean cumulative radon exposure (WLM)*	Mean annual exposure rate (WL)*
*Full* *cohort* (Rage et al. 2020, Tables 1 and 2)						
Eldorado (Canada)	1950–1999	13,574	517	2	122	8.3
Ontario (Canada)	1954–2007	28,546	1246	5	31	0.9
Czech (Czech Rep.)	1952–2014	9978	1176	8	73	0.8
France (France)	1946–2007	5086	213	17	37	0.8
Colorado (USA)	1960–2005	4137	612	4	579	11.7
New Mexico (USA)	1957–2012	3469	231	9	90	9.6
Wismut (Germany)	1946–2013	54,919	3759	14	304	1.9
PUMA total		119,709	7754	10	191	2.9
PUMA without Wismut		64,790	3995	6	98	3.7
*1960 + sub-cohort*^#^ (Richardson et al. 2022, Tables 1 and 2)						
Eldorado (Canada)	1960–1999	6593	91	2	7	0.2
Ontario (Canada)	1960–2007	15,810	299	6	6	0.4
Czech (Czech Rep.)	1960–2014	5532	228	6	7	0.2
France (France)	1960–2007	2159	19	17	12	0.1
Colorado (USA)	1960–2005	175	16	2	193	7.5
New Mexico (USA)	1960–2012	2537	94	9	39	4.7
Wismut (Germany)	1960–2013	25,067	470	10	18	0.3
PUMA total		57,873	1217	8	13	0.5
PUMA without Wismut		32,806	747	6	10	0.7

More detailed information can be found in Rage et al. (2020) and Richardson et al. (2022)

*WLM* Working level months; *WL* Working level

*Non-exposed miners (i.e., with WLM = 0) were excluded from calculation of mean values

^#^miners hired in 1960 or later

### Statistical methods

Lifetime risks reflect the probability of developing or dying from a specific disease of interest (here: lung cancer mortality) in the course of a lifetime. The lifetime excess absolute risk (LEAR) is defined as the difference between the lifetime risk ${\text{LR}}_E$ for an individual with exposure $E$ (here: exposure to occupational radon) and the lifetime risk ${\text{LR}}_0$ for an individual without exposure

$$\begin{aligned} {\text{LEAR}} & = {\text{LR}}_E - {\text{LR}}_0 \\ & = \mathop \int \limits_0^\infty r_E \left( a \right)S\left( a \right)da - \mathop \int \limits_0^\infty r_0 \left( a \right)S\left( a \right)da, \\ \end{aligned}$$

with survival function $S\left( a \right) = e^{ - \mathop \smallint \limits_0^a q_0 \left( u \right)du}$ describing the probability to survive until age $a$, and baseline mortality rates for all causes of death $q_0 \left( a \right)$ and for lung cancer $r_0 \left( a \right)$ at age $a$ in absence of exposure. The lung cancer mortality rate $r_E (a)$ at age $a$ under exposure is assumed to follow the typical general model structure $r_E (a)$ = $r_0 \left( a \right)\left( {1 + {\text{ERR}}\left( a \right)} \right)$ with excess relative risk term ${\text{ERR}}\left( a \right)$. Based on this assumption, the LEAR can be approximated and finally technically calculated by

$$\begin{aligned} {\text{LEAR}} & \approx \mathop \sum \limits_{a = 0}^{a_{\max } } r_E \left( a \right)\tilde{S}(a) - \mathop \sum \limits_{a = 0}^{a_{\max } } r_0 \left( a \right)\tilde{S}(a) \\ & = \mathop \sum \limits_{a = 0}^{a_{\max } } r_0 \left( a \right){\text{ERR}}\left( a \right)\tilde{S}\left( a \right), \\ \end{aligned}$$

where $\tilde{S}(a) = e^{ - \mathop \sum \limits_{u = 0}^{a - 1} q_0 (u)}$ approximates the survival function $S\left( a \right)$. The ${\text{ERR}}\left( a \right)$ depends on an exposure pattern and a specific risk model, e.g., with a structure as in the BEIR VI exposure–age–concentration model, and a lag time. The final summary result is reported as the LEAR per WLM, obtained by dividing the calculated LEAR by the cumulative exposure accrued over the entire exposure scenario (here: 94 WLM). For example, an LEAR for lung cancer mortality per WLM of 5 × 10^–4^ means that among 100 people with a cumulative occupational radon exposure of 100 WLM five additional (excess) lung cancer deaths would occur due to this exposure during lifetime.

### Lag time

A lag is assumed between exposure to radon and any observed change in the lung cancer mortality rate. In the risk models employed here, the lag is either directly described by the model structure (e.g., BEIR VI exposure–age–concentration model with ${\text{ERR}}(a) = 0$ for ${\text{TSE}}(a) < 5$) or by the data grouping process prior to any model fit as for the parametric risk models with continuous effect modifying variables (e.g., for the Czech/French cohort, Tomasek et al. 2008b). In that analysis, a miner’s exposure is lagged a priori by $L = 5$ years. A lag assumption may be incorporated in the LEAR calculation by calculating ${\text{ERR}}(a)$ at age $a$ only with information about radon exposure until age $a - L$. However, doing so would violate the important equation $a = {\text{AME}}(a) + {\text{TSME}}(a)$, with ${\text{AME}}$ being the time-varying age at median exposure and ${\text{TSME}}$ the time since median exposure. This is technically solved by considering ${\text{AME}}(a - L)$ and ${\text{TSME}}\left( {a - L} \right) + L$ in the calculation of ${\text{ERR}}(a)$.

### LEAR calculations

For the calculation of LEAR, the maximum age was set to $a_{\max } = 94$, i.e., the LEAR was calculated up to age < 95 years. The baseline lung cancer mortality rates $r_0 (a)$ and all-cause mortality rates $q_0 (a)$ were taken from the ICRP mixed Euro-American-Asian population (ICRP 2007) to be comparable with previous publications (UNSCEAR 2020, Tomasek et al. 2008b). According to UNSCEAR (2020) and other LEAR calculations (ICRP 2010, Tomasek et al. 2008b), the exposure scenario was defined as 2 WLM per year from age 18 to 64 years with a lag of $L = 5$ years.

To compare LEAR estimates for mortality from lung cancer of the PUMA study with those from previous studies, the LEAR per WLM for all published risk models of uranium miners studies that include time- and age-related effect modifiers have been re-estimated, while using the same exposure scenario, baseline rates, and survival function. For this reason, some estimates may slightly differ from previously published LEAR values. The coefficients describing the relative risk model were the values as reported in the original papers, and are described in Tables 2 and 3 for the PUMA study and in Supplementary Tables 1–3 for other studies. The LEAR for the complete exposure scenario (i.e., 2 WLM per year from age 18 to 64 years, resulting in a cumulative exposure of 94 WLM) can be obtained by multiplying the value for the LEAR per WLM with 94. All LEAR calculations were performed with the statistical software R (R Core Team 2022).

**Table 2:** Lifetime excess absolute risk (LEAR) estimates obtained using a model with effect modifiers defined by categories of time since exposure, attained age, and annual exposure rate (BEIR VI exposure–age–concentration model) in the full PUMA cohort and the PUMA 1960 + sub-cohort

	PUMA full cohort Kelly-Reif et al. (2023)		PUMA 1960 + sub-cohort Richardson et al. (2022)	
	Lung cancer deaths (*n*)	Estimate (95% CI)	Lung cancer deaths (*n*)	Estimate (95% CI)
ERR/100 WLM	7754	4.68 (2.88, 6.96)	1217	6.98 (1.97, 16.15)
*Time* *since* *exposure* *(years)*				
5–14		1.0		1.0
15–24		0.77 (0.56, 1.05)		0.64 (0.17, 2.43)
25–34		0.54 (0.38, 0.76)		0.89 (0.34, 3.01)
35 +		0.39 (0.26, 0.58)		–
*Attained* *age* *(years)*				
< 55	1380	1.0	302	1.0
55–64	2568	0.55 (0.38, 0.82)	490	0.64 (0.25, 1.68)
65–74	2640	0.38 (0.25, 0.57)	351	0.22 (0.06, 0.67)
75 +	1166	0.40 (0.24, 0.66)	74	0.17 (n.d., 0.85)
*Annual* *exposure* *rate* *(WL)*				
< 0.5		1.0		1.0
0.5–1.0		0.60 (0.31, 1.08)		1.00 (0.38, 2.36)
1.0–5.0		0.42 (0.31, 0.64)		0.29 (0.11, 0.68)
5.0 +		0.17 (0.12, 0.25)		–
LEAR per WLM (× 10^4^)		5.38		7.50

*ERR* Excess relative rate; *CI* Confidence interval; *LEAR* Lifetime excess absolute risk; *PUMA* Pooled uranium miners analysis; *WLM* Working level months, *WL* Working level; *n.d.* Lower bound not determined

**Table 3:** Lifetime excess absolute risk (LEAR) estimates obtained using a model with effect modifiers defined by categories of age at exposure, attained age, and annual exposure rate in the full PUMA cohort and the PUMA 1960 + sub-cohort

	PUMA full cohort Kelly-Reif et al. (2023)		PUMA 1960 + sub-cohort Richardson et al. (2022)	
	Lung cancer deaths (*n*)	Estimate (95% CI)	Lung cancer deaths (*n*)	Estimate (95% CI)
ERR/100 WLM	7754	6.47 (3.39, 10.06)	1217	8.38 (3.30, 18.99)
*Age* *at* *exposure* *(years)*				
50 +		1.0		1.0*
35–49		0.83 (0.54, 1.39)		1.0*
< 35		0.55 (0.36, 0.92)		0.59 (0.30, 1.20)
*Attained* *age* *(years)*				
< 55	1380	1.0	302	1.0
55–64	2568	0.40 (0.28, 0.59)	490	0.55 (0.24, 1.30)
65–74	2640	0.21 (0.15, 0.31)	351	0.20 (0.06, 0.53)
75 +	1166	0.19 (0.12, 0.29)	74	0.14 (n.d., 0.64)
*Annual* *exposure* *rate* *(WL)*				
< 0.5		1.0		1.0
0.5–1.0		0.57 (0.29, 1.00)		1.23 (0.49, 2.77)
1.0–5.0		0.39 (0.28, 0.58)		0.33 (0.13, 0.75)
5.0 +		0.15 (0.11, 0.22)		-
LEAR per WLM (× 10^4^)		5.57		7.66

*ERR* Excess relative rate; *CI* Confidence interval; *LEAR* Lifetime excess absolute risk; *PUMA* Pooled uranium miners analysis; *WLM* Working level months, *WL* Working level; *n.d.* Lower bound not determined

*Reference category is ≥ 35 years (i.e., categories 50 + and 35–49 years are combined)

## Results

**Fig. 1:**
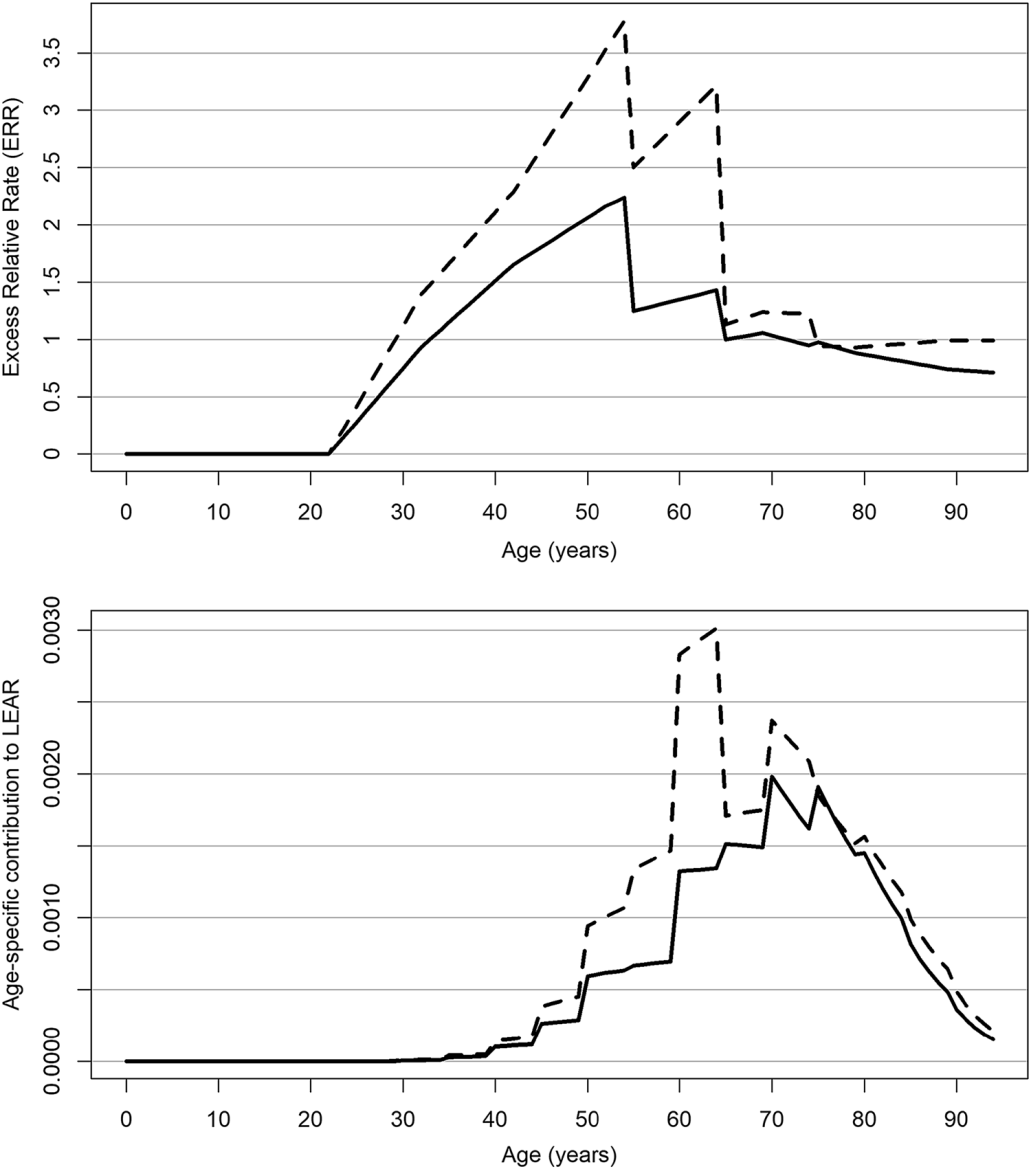
LEAR components by attained age (Upper part: ${\text{ERR}}(a)$, Bottom part: age-specific contribution to LEAR, $r_0 \left( a \right){\text{ERR}}(a)\tilde{S}(a)$) predicted in the full PUMA cohort (Kelly-Reif et al. 2023, solid line) and the PUMA 1960 + sub-cohort (Richardson et al. 2022, dashed line) for the exposure scenario of 2 working level months (WLM) per year from age 18 to 64 up to age < 95 years, assuming a 5-year lag for the BEIR VI exposure–age–concentration model, and using baseline mortality rates derived from the ICRP mixed Euro-American-Asian population (ICRP 2007)

Table 2 shows the radon-related lung cancer risk in the full PUMA cohort and in the PUMA 1960 + sub-cohort based on the BEIR VI exposure–age–concentration model with categorical effect modifiers time since exposure, attained age, and annual exposure rate. The ERR/100 WLM at attained age < 55 years, 5–14 years since exposure, and exposure rate < 0.5 WL was 4.68 (95% CI: 2.88, 6.96) and 6.98 (95% CI: 1.97, 16.15) in the full cohort (Kelly-Reif et al. 2023) and 1960 + sub-cohort (Richardson et al. 2022), respectively. The estimated ERR/100 WLM decreased with increasing attained age, radon exposure rate and time since exposure, the latter decrease, however, is only present in the full cohort and not the 1960 + sub-cohort. The estimated LEAR per WLM is slightly higher in the 1960 + sub-cohort compared with the full cohort (7.50 × 10^*−*4^ vs 5.38 × 10^*−*4^, respectively). This is also illustrated in Fig. 1 (upper part) where the ${\text{ERR}}(a)$ is plotted as a function of attained age, $a$, under the exposure scenario of interest (i.e., 2 WLM per year from age 18 to 64 years). Notably, using the model coefficients derived for the 1960 + sub-cohort, the ERR/100 WLM increases slightly after age 75 years, which is mainly due to the value of the parameter estimate for the effect modifier time since exposure. The estimated value of the coefficient for this modifier was highest for the category 5–14 years after exposure (reference category 1.0), decreased for the category 15–24 years after exposure to 0.64, and increased again for the category 25 years or more after exposure to 0.89. The bottom part of Fig. 1 shows the corresponding age-specific contribution to LEAR, $r_0 \left( a \right){\text{ERR}}(a)\tilde{S}(a)$ for each age $a$. Within the full PUMA cohort, the largest LEAR contribution is observed at ages 70–75 years, which is 5–10 years after the maximum cumulative exposure is reached. From age 75 years onwards, there is a strong decrease in the age-specific contribution to the LEAR which reflects the decreasing baseline lung cancer mortality rates $r_0 \left( a \right)$, the decrease in ${\text{ERR}}(a)$ with increasing time since exposure, and the decreasing fraction of the cohort who remains at risk of lung cancer (Supplementary Fig. 1). For the PUMA 1960 + sub-cohort, a similar pattern is observed; however, the peak in the contribution to LEAR is between 60 and 65 years, thus 10 years earlier than in the full PUMA cohort.

Table 3 presents the LEAR per WLM based on an alternative risk model for the PUMA study with categorical effect modifiers age at exposure, attained age, and exposure rate (i.e., the BEIR VI model, but with age at exposure instead of time since exposure). The ERR/WLM decreases with increasing attained age and increases with increasing age at exposure in both cohorts. The corresponding LEAR per WLM is 7.66 × 10^*−*4^ in the 1960 + sub-cohort and 5.57 × 10^*−*4^ in the full cohort, respectively, and thus comparable to that based on the BEIR VI exposure–age–concentration model. Again, the estimated LEAR is higher in the 1960 + sub-cohort compared to the full cohort. Supplementary Fig. 2 (upper part) shows that the ${\text{ERR}}(a)$ is highest at younger attained ages, however no further decrease in ${\text{ERR}}(a)$ is observed after age 65 years in the full cohort or after age 75 years in the 1960 + sub-cohort. Supplementary Fig. 2 (bottom part) provides a similar pattern as for the BEIR VI model. Again, in the full PUMA cohort, there is an increase in the age-specific contribution to LEAR up to age 70–75 years and then a strong decrease. This corresponding peak in the 1960 + sub-cohort is again at ages 60–65 years.

To compare the LEAR per WLM of the PUMA study with those estimated in the previous studies, the LEAR per WLM for all published risk models of uranium miners studies that include time- and age-related effect modifiers have been re-estimated. Table 4 provides an overview of these studies, their characteristics, and related LEAR per WLM. The very first published study providing a relative risk model was a meta-analysis of 7 cohorts, the so-called “Jacobi study” (ICRP 1993; Chmelevsky et al. 1994) with a re-estimated LEAR per WLM of 3.20 × 10^*−*4^ based on 1047 lung cancer deaths and 31,486 miners. The cohort included a wide range of exposures and exposure rates; however, risk models did not account for exposure rate. This may have introduced an underestimation of true risk at low exposures due to ignoring the well-established inverse exposure-rate effect (NRC 1999, UNSCEAR 2020). In 1999, the results of the pooled analyses of the 11 miners cohort study were published (NRC 1999), including more than twice the number of miners (*n* = 67,897) and three times more lung cancer deaths (*n* = 2787) than the Jacobi study. In addition, as a new model, the BEIR VI exposure–age–concentration model was developed and applied (NRC 1999). For this risk model, the estimated LEAR per WLM was 5.97 × 10^*−*4^ and thus two times higher compared to the Jacobi study.

**Table 4:** Lifetime excess absolute risk (LEAR) estimates obtained using previously published risk models of uranium miners cohort studies that include time- and age-related effect modifiers

Study	Publication	# Miners	# Lung cancer deaths	Person-years at risk (Mio)	Mean WLM	Mean duration of follow-up	Mean duration of employment	Model	LEAR per WLM (× 10^4^)
Meta estimate from 7 miners cohorts	ICRP (1993)	31,486	1047	0.6	120	n.a	n.a	Cat. Model (TSE, AA)	3.20
*Full cohorts, full range of exposures, three modifiers TSE or AE, AA, and ER*									
Pooled 11 miners cohort	NRC (1999)	67,897	2787	1.2	164	18	6	BEIR VI (TSE, AA, ER)	5.97
Eldorado	Lane et al. (2010)	16,236	618	0.5	117	31	2	BEIR VI (TSE, AA, ER)	8.20
Wismut (FU 2013)	Kreuzer et al. (2018)	58,974	3942	2.3	280	40	13	BEIR VI (TSE, AA, ER)	2.50
Czech cohort	UNSCEAR (2020)	9978	1141	0.3	73	31	8	BEIR VI (TSE, AA, ER)	4.22
Wismut (FU 2018)	Kreuzer et al. (2023)	58,972	4329	2.5	280	42	13	BEIR VI (TSE, AA, ER)	3.13
PUMA	Kelly-Reif et al. (2023)	119,709	7754	4.3	191	36	10	BEIR VI (TSE, AA, ER)	5.38
PUMA	Kelly-Reif et al. (2023)	119,709	7754	4.3	191	36	10	Cat. Model (AE, AA, ER)	5.57
PUMA without Wismut	Kelly-Reif et al. (2023)	64,790	3995	2.2	98	33	6	BEIR VI (TSE, AA, ER)	8.78
*Cohorts restricted to more recent periods with chronic low exposures or exposure rates, three modifiers TSE or AE, AA, and ER*									
Cz + Fr (measured)*	Tomasek et al. (2008a)	10,100	547	0.2	47	n.a	n.a	Cont. Model (TSME, AME)	4.58
Cz + Fr + Eldorado (< 100 WLM)	Lane et al. (2019)	n.a	408	0.4	36	n.a	n.a	BEIR VI (TSE, AA, ER)	4.56
Wismut 1960 + (FU 2013)	Kreuzer et al. (2023)	26,764	495	1.0	17	36	10	BEIR VI (TSE, AA, ER)	9.22
Wismut 1960 + (FU 2018)	Kreuzer et al. (2023)	26,764	663	1.1	17	40	10	BEIR VI (TSE, AA, ER)	6.10
PUMA 1960 +	Richardson et al. (2022)	57,873	1217	1.9	13	33	8	BEIR VI (TSE, AA, ER)	7.50
PUMA 1960 +	Richardson et al. (2022)	57,873	1217	1.9	13	33	8	Cat. Model (AE, AA, ER)	7.66

LEAR calculation based on: Exposure scenario: 2 WLM per year from age 18 to 64 years, Baseline mortality rate: mixed Euro-American-Asian population (ICRP 2007), Lifetime risk calculated up to age < 95 years, Projection model: relative risk model

*TSE* Time since exposure; *AA* Attained age; *AE* Age at exposure; *ER* Exposure rate; *TSME* Time since median exposure; *AME* Age at median exposure; *LEAR* Lifetime excess absolute risk; *WLM* Working level months; *BEIR VI*: BEIR VI exposure–age–concentration model (NRC 1999); *n.a.* Not available

*Continuous variables for the effect modifiers time since median exposure (TSME) and age at median exposure (AME) restricted to person-years at risk with “measured” (annual estimates based on current ambient measurements or personal dosimeters) instead of “estimated” (retrospectively estimated or extrapolated) radon concentrations

The BEIR VI pooled analysis did not include the newly established large German Wismut cohort (Grosche et al. 2006). The full Wismut cohort comprises 58,974 workers and 3942 lung cancer deaths at end of follow-up in 2013 (Kreuzer et al. 2018) and resulted in an LEAR per WLM of 2.50 × 10^*−*4^. With the extended mortality follow-up to end of 2018 and additional baseline stratification by duration of employment like in the PUMA cohort, the LEAR per WLM increased to 3.13 × 10^*−*4^ (Kreuzer et al. 2023). Two smaller individual studies, the Czech (UNSCEAR 2020) and Eldorado (Lane et al. 2010) cohorts, showed LEAR estimates per WLM of 4.22 × 10^*−*4^ and 8.20 × 10^*−*4^, respectively. The PUMA full cohort is currently the largest study with 119,709 miners and 7754 lung cancer deaths and integrates most of the updated studies included in BEIR VI and the Wismut cohort. The estimated LEAR per WLM of 5.38 × 10^*−*4^ or 5.57 × 10^*−*4^ (depending on choice of model) is consistent with that of the BEIR VI study and two times higher than that for the full Wismut cohort.

Table 4 additionally provides information on cohorts restricted to chronic low exposures and exposure rates. The estimated LEAR per WLM was around 4.6 × 10^*−*4^ for two smaller studies, the pooled analyses of full Czech and French cohorts with restriction of person-years at risk to measured radon exposure (Tomasek et al. 2008a) and of the Czech, French and Eldorado sub-cohorts with restriction to more recent years and exposures less than 100 WLM (Lane et al. 2019). The LEAR in the Wismut 1960 + sub-cohort with end of follow-up 2013 and 2018 (Kreuzer et al. 2023) were 9.22 × 10^*−*4^ and 6.10 × 10^−4^, respectively. Among these low exposure/exposure-rate studies, the PUMA 1960 + sub-cohort is by far the largest study (57,873 miners and 1217 lung cancer deaths) and involves the lowest average radon exposure (13 WLM), the corresponding LEAR was around 7.50 × 10^*−*4^. Compared to the respective full cohorts, the LEAR of the 1960 + sub-cohorts of the PUMA and the Wismut study were somewhat higher.

## Discussion

PUMA provides the largest and most informative database to date to estimate the risk of death from lung cancer due to cumulative radon exposure in studies of uranium miners. The LEAR per WLM is estimated to lie between 5.38 × 10^*−*4^ and 7.66 × 10^*−*4^ depending on the choice of model and the use of the full cohort or the 1960 + sub-cohort with a focus on more recent periods of chronic low exposure. While the choice of model within a given cohort has a nearly negligible effect on the resulting LEAR, the consideration of either the full cohort or 1960 + sub-cohort makes a difference, with somewhat higher LEAR results for the latter cohort. In contrast to the PUMA full cohort, in the 1960 + sub-cohort the estimated parameters of the relative risk model have less heterogeneity between studies, but wider confidence intervals.

### Comparison of results from full and 1960 + sub-cohorts

In the full PUMA cohort, heterogeneity in risk estimates between studies has been reported by Kelly-Reif et al. (2023), which was in part attributed to the Wismut study, which forms half of the data of PUMA (2.2 out of 4.3 million person-years at risk). The PUMA full cohort excluding the Wismut study would result in an LEAR per WLM of 8.78 × 10^*−*4^. This restricted cohort differs from the PUMA Wismut cohort in some characteristics, e.g., appreciably lower exposures and shorter duration of employment (see also Table 1). For example, within the full PUMA study, 82% of the person-years at risk accrued from radon exposures above 250 WLM and about 70% of all person-times at risk with duration of employment more than 10 years are from the Wismut cohort (Kelly-Reif et al. 2023 Suppl. Table 1), respectively. It is unclear whether this difference has some influence on the risk estimates. The overview on LEAR estimates from published uranium miners studies in Table 4 shows that the findings of the full Wismut cohort are at the lower end of the range of all calculated LEAR. Possible reasons for this observed lower estimated risk like competing risk of silicosis, measurement error in exposure assessment, or possibly incomplete follow-up in the very early years (1946–1960) were addressed in detail in Kreuzer et al. (2023).

In contrast to the analyses based on full cohorts (Kelly-Reif et al. 2023), PUMA analyses of the 1960 + sub-cohorts did not provide any evidence of heterogeneity in risk estimates between studies (Richardson et al. 2022). The 1960 + sub-cohorts allow direct estimation of health effects of chronic exposure to low radon concentrations at low exposure rates which is of interest for radiation protection today. It also allows to exclude miners with extreme levels of exposure (estimated effective doses for some miners employed in the early years could reach several hundreds or thousands of mSv per year) (Laurier 2020). In addition, no complex modeling of exposure rate is necessary as compared to the full cohort; in several of the component studies, exposure rates were one or two orders of magnitude higher in the early years compared to 1960 or later. Furthermore, exposure assessment in these later years was often based on measurements rather than on expert rating. A higher quality of exposure assessment decreases measurement error and thus the potential for underestimation of risk. However, the 1960 + sub-cohorts involve lower statistical power due to smaller size, high uncertainty in parameter estimates, shorter duration of follow-up, and younger age compared to the full cohorts. The observed increase in ERR at older ages in the PUMA 1960 + sub-cohort (Fig. 1 upper part) and particularly in the Wismut 1960 + sub-cohort with end of follow-up 2013 (Kreuzer et al. 2023, Supplementary Fig. 3) seems implausibly high. In these young 1960 + sub-cohorts, lung cancer deaths are still rare at ages over 75 years and at more than 35 years since exposure (see Tables 2 and 3). Consequently, it is likely that the decrease in ERR/WLM with increasing time since exposure and attained age cannot be completely described by the data of 1960 + sub-cohorts. For example, in the Wismut 1960 + sub-cohort, the extension of end of follow-up from 2013 to 2018 led to a decrease of LEAR per WLM from 9.22 to 6.10 × 10^*−*4^ (Kreuzer et al. 2023 Suppl. Table 3). Thus, further follow-up of individual PUMA studies will allow refining risk estimates derived from 1960 + cohorts in the future.

### Strengths and limitations

The current calculations of the LEAR for lung cancer due to radon from various uranium miners studies offer several strengths. First, similar methods have been used, and thus, LEAR values based on different studies and relative risk models are directly comparable. Second, for the first time, LEAR was calculated based on the worldwide largest and most informative study PUMA. More than 4.3 million person-years at risk and nearly 8000 lung cancer deaths with a long duration of follow-up form the basis for PUMA (Rage et al. 2020; Richardson et al. 2021, 2022; Kelly-Reif et al. 2023). This large database allows—in contrast to many individual studies—for detailed consideration of relevant effect modifiers age, time since exposure and exposure rate in the risk model, a recommendation that was recently reinforced by UNSCEAR (2020). Third, the LEAR were determined for cohorts restricted to low exposures and exposure rates including all three effect modifiers in the risk models.

A limitation of the current LEAR analyses is that many factors with potential influence on the LEAR have not yet been evaluated. This concerns (1) the use of different and more suitable baseline mortality rates as well as evaluation of effects of the increasing survival trend for lung cancer, (2) consideration of smoking (interaction of smoking with radon, change of smoking patterns over time), (3) application of other scenarios from occupational or residential radon exposure, (4) consideration of annual instead of average exposure rates in risk models (Tomasek 2020), (5) use of different risk projection models (relative/additive/mixed), and (6) evaluation of uncertainties associated with LEAR estimates (e.g., confidence intervals). A general limitation of all the uranium miners studies considered in this paper is that they include only men and that only mortality and no incidence data for lung cancer are available.

## Conclusion

PUMA clearly strengthens evidence on the shape of the exposure–response relationship between radon exposure and lung cancer mortality in uranium miners and thus the estimation of the LEAR. The range of currently available LEAR values for lung cancer at low exposures and exposure rates derived from different models and previous publications based on smaller studies is 2.5 to 9.2 × 10^–4^ per WLM, with the current PUMA findings (5.4 up to 7.7 × 10^*−*4^ per WLM) being in the upper half of this range. Continued mortality follow-up of the studies included in PUMA, particularly of the 1960 + sub-cohorts, is expected to provide additional insights and is therefore strongly recommended.

### Supplementary Information

Below is the link to the electronic supplementary material.

Supplementary file1 (DOCX 137 KB)
