# Supplementary material for: Lifetime excess absolute risk for lung cancer due to exposure to radon: results of the pooled uranium miners cohort study PUMA
Source: Radiat Environ Biophys. 2024 Jan 3;63(1):7–16. doi: 10.1007/s00411-023-01049-w (PMC10920468; doi:10.1007/s00411-023-01049-w)
Supplement: Supplementary file 1 — Supplementary file1 (DOCX 137 KB) [file 411_2023_1049_MOESM1_ESM.docx]

**Supplementary Table 1: Lifetime excess absolute risk (LEAR) estimates obtained from uranium miners studies using the BEIR VI exposure-age-concentration model, full cohorts with full range of exposure**

|  | **11 miners cohort** *NRC 1999* | | **Czech cohort** *UNSCEAR 2020* | **Eldorado** *Lane et al. 2010* | **Wismut (FU 2013)** *Kreuzer et al. 2018* | **Wismut (FU 2018)** *Kreuzer et al. 2023* | **PUMA** *Kelly-Reif et al. 2023* | **PUMA without Wismut** *Kelly-Reif et al. 2023* |
| --- | --- | --- | --- | --- | --- | --- | --- | --- |
| Lung cancer deaths | | **2,774** | **1,141** | **618** | **3,942** | **4,329** | **7,754** | **3,995** |
| Person-years at risk | | **1.2 Mio** | **0.3 Mio** | **0.5 Mio** | **2.3 Mio** | **2.5 Mio** | **4.3 Mio** | **2.2 Mio** |
|  | |  |  |  |  |  |  |  |
| ERR/100 WLM (95% CI) | | 7.68 | 6.47 | 6.11 (1.51, 17.82) | 2.31 (1.20, 4.13) | 2.50 (0.81, 4.18) | 4.68 (2.88, 6.96) | 6.91 (3.82; 11.3) |
| Time since exposure (years) | |  |  |  |  |  |  |  |
| 5-14 | | 1.0 | 1.0 | 1.0 | 1.0 | 1.0 | 1.0 | 1.0 |
| 15-24 | | 0.78 | 0.77 | 0.47 (p<0.001) | 0.79 (0.48, 1.36) | 0.96 (0.47, 1.46) | 0.77 (0.56, 1.05) | 0.70 (0.47; 0.98) |
| 25-34 | | 0.51 | 0.41 | 0.29 (p<0.001) | 0.54 (0.33, 0.91) | 0.64 (0.30, 0.97) | 0.54 (0.38, 0.76) | 0.45 (0.27; 0.66) |
| 35+ | | - | - | - | 0.52 (0.32, 0.90) | 0.61 (0.27, 0.94) | 0.39 (0.26, 0.58) | 0.27 (0.14; 0.45) |
| Attained age (years) | |  |  |  |  |  |  |  |
| <55 | | 1.0 | 1.0 | 1.0 | 1.0 | 1.0 | 1.0 | 1.0 |
| 55-64 | | 0.57 | 0.28 | 1.62 (0.57, 4.59) | 0.48 (0.31, 0.74) | 0.44 (0.27, 0.70) | 0.55 (0.38, 0.82) | 0.81 (0.49; 1.48) |
| 65-74 | | 0.29 | 0.22 | 0.82 (0.27, 2.52) | 0.33 (0.21, 0.52) | 0.33 (0.20, 0.55) | 0.38 (0.25, 0.57) | 0.51 (0.29; 1.02) |
| 75+ | | 0.09 | 0.21 | 0.19 (0.01, 2.38) | 0.32 (0.19, 0.55) | 0.34 (0.19, 0.60) | 0.40 (0.24, 0.66) | 0.50 (0.20; 1.16) |
| Average or annual exposure rate (WL) | |  |  |  |  |  |  |  |
| <0.5 | | 1.0 | 1.0 | 1.0 | 1.0 | 1.0 | 1.0 | 1.0 |
| 0.5-1.0 | | 0.49 | 0.63 | 1.05 (0.40, 2.80) | 0.61 (0.36, 1.01) | 0.60 (0.36, 0.99) | 0.60 (0.31, 1.08) | 0.44 (0.21; 0.83) |
| 1.0-3.0 | | 0.37 | 1.29 | 0.47 (0.19, 1.18) | 0.44 (0.29, 0.68) | 0.40 (0.26, 0.61) | 0.42 (0.31, 0.64) | 0.41 (0.29; 0.64) |
| 3.0-5.0 | | 0.32 | - | 0.34 (0.11, 1.01) | 0.39 (0.25, 0.59) | 0.34 (0.22, 0.53) | 0.42 (0.31, 0.64) | 0.14 (0.09; 0.23) |
| 5.0-15.0 | | 0.17 | - | 0.31 (0.12, 0.81) | 0.34 (0.22, 0.52) | 0.28 (0.18, 0.44) | 0.17 (0.12, 0.25) | - |
| 15.0+ | | 0.11 | - | 0.16 (0.06, 0.43) | 0.16 (0.08; 0.32) | 0.15 (0.08, 0.26) | - | - |
|  | |  |  |  |  |  |  |  |
| **LEAR per WLM (×10^4^)** | | **5.97** | **4.22** | **8.20** | **2.50** | **3.13** | **5.38** | **8.78** |

ERR: Excess relative rate, CI: Confidence interval
LEAR: Lifetime excess absolute risk
PUMA: Pooled uranium miners analysis
WLM: Working level months, WL: Working level
FU: Follow-up

**Supplementary Table 2: Lifetime excess absolute risk (LEAR) estimates obtained from uranium miners studies using the BEIR VI exposure-age-concentration model in cohorts restricted to more recent periods with low exposures or exposure rates**

|  | **Czech/French/Eldorado** *Lane et al. 2019* | **PUMA 1960+ sub-cohort** *Richardson et al. 2022* | **Wismut 1960+ sub-cohort (FU 2013)** *Kreuzer et al. 2023* | **Wismut 1960+ sub-cohort (FU 2018)** *Kreuzer et al. 2023* |
| --- | --- | --- | --- | --- |
| Lung cancer deaths | **408** | **1,217** | **495** | **663** |
| Person-years at risk (million) | **0.4** | **1.9** | **1.0** | **1.1** |
|  |  |  |  |  |
| ERR/100 WLM (95% CI) | 5.6 (2.0, 13.3) | 6.98 (1.97, 16.15) | 7.13 (<0, 16.84) | 6.92 (<0, 16.59) |
| Time since exposure (years) |  |  |  |  |
| 5-14 | 1.0 | 1.0 | 1.0 | 1.0 |
| 15-24 | 0.89 (0.45, 1.74) | 0.64 (0.17, 2.43) | 0.79 (<0, 2.03) | 0.95 (<0, 2.40) |
| 25+ | 0.38 (0.14, 0.78) | 0.89 (0.34, 3.01) | 0.35 (<0, 0.88) | 0.36 (<0, 0.92) |
| Attained age (years) |  |  |  |  |
| <55 | 1.0 | 1.0 | 1.0 | 1.0 |
| 55-64 | 0.78 (0.29, 2.10) | 0.64 (0.25, 1.68) | 0.90 (0.25, 3.18) | 0.83 (0.24, 2.84) |
| 65-74 | 0.37 (0.04, 1.26) | 0.22 (0.06, 0.67) | 0.42 (0.09, 1.97) | 0.34 (0.08, 1.52) |
| 75+ | 0.02 (n.a.) | 0.17 (n.d., 0.85) | 0.49 (0.04, 5.62) | 0.09 (0.01, 5.89) |
|  |  |  |  |  |
| Exposure rate* (WL) |  |  |  |  |
| <0.5 | 1.0 | 1.0 | 1.0 | 1.0 |
| 0.5-1.0 | 0.83 (0.48, 1.66) | 1.00 (0.38, 2.36) | 0.85 (0.46, 1.57) | 0.90 (0.50, 1.64) |
| 1.0+ | 2.00 (0.44, 5.86) | 0.29 (0.11, 0.68) | 0.31 (0.06, 1.53) | 0.57 (0.20, 1.62) |
|  |  |  |  |  |
| **LEAR per WLM (×10^4^)** | **4.56** | **7.50** | **9.22** | **6.10** |

* Exposure rate: time-varying annual exposure rate in PUMA, time-varying average exposure rate in Wismut models

FU: Follow-up
ERR: Excess relative rate, CI: Confidence interval
LEAR: Lifetime excess absolute risk
PUMA: Pooled uranium miners analysis
WLM: Working level months, WL: Working level

**Supplementary Table 3: Lifetime excess absolute risk (LEAR) estimates in the pooled analyses of full cohorts of the Czech and French cohorts (Tomášek et al. 2008a) restricted to person-years at risk of measured radon values**

| Parameter | Estimate | 95% Confidence interval |
| --- | --- | --- |
| ERR/WLM (Measured) | 0.042 | 0.024, 0.072 |
| ERR/WLM (Estimated) | 0.009 | 0.001, 0.024 |
| Time since median exposure^a^ | 0.45 | 0.28, 0.70 |
| Age at median exposure^b^ | 0.52 | 0.32, 0.81 |
| **LEAR per WLM (×10^4^)** | **4.58** |  |

For the LEAR calculation, the ERR/WLM for measured radon concentrations was used
a: Relative effect per 10 years of time since median exposure
b: Relative effect per 10 years of age at median exposure
ERR: Excess relative rate
LEAR: Lifetime excess absolute risk
WLM: Working level months


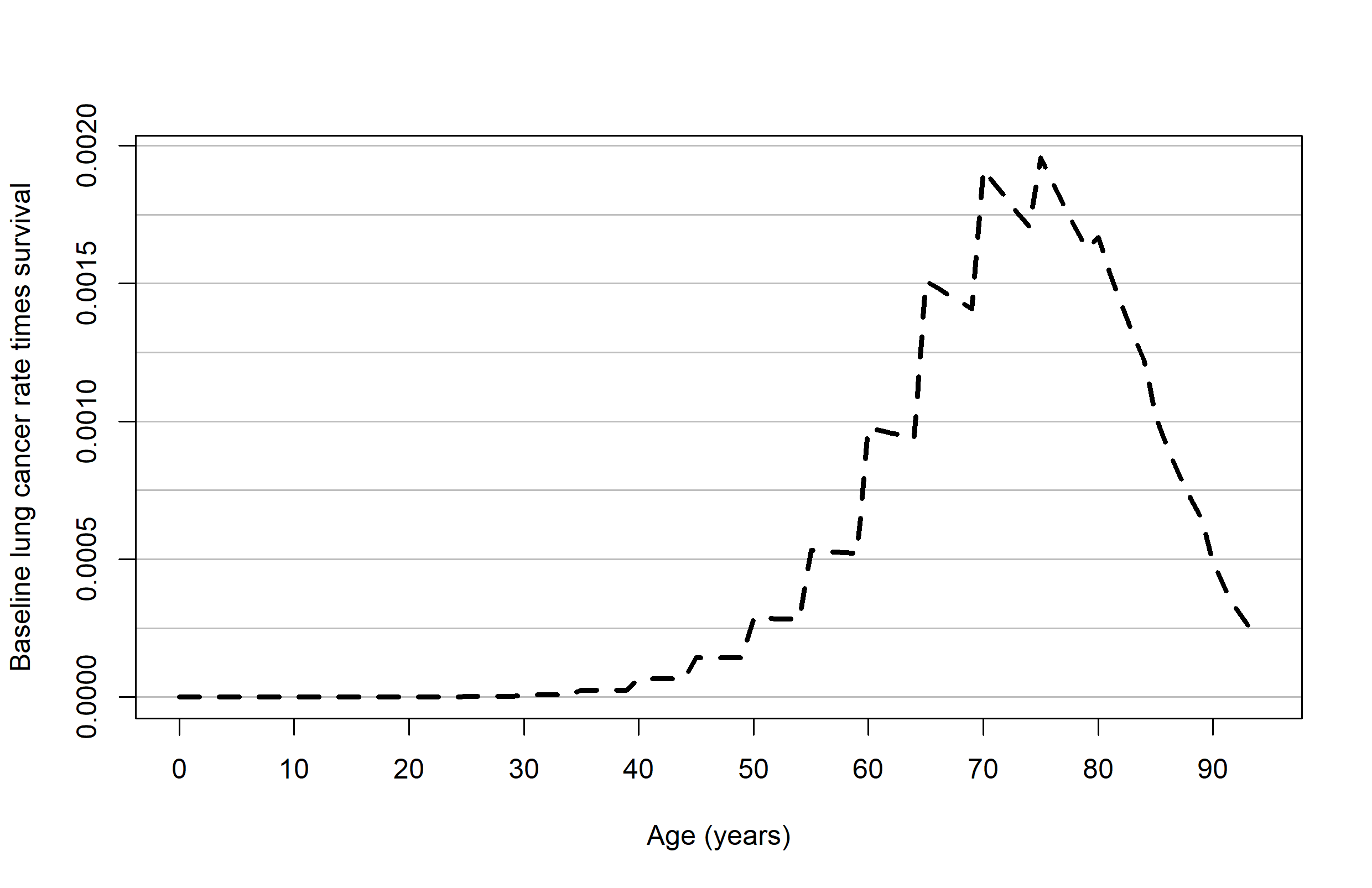


**Suppl. Figure 1:** Product of baseline lung cancer mortality rate from mixed Euro-American-Asian population (ICRP 2007) and survival probability, $r_{0}\left( a \right)\tilde{S}\left( a \right)$ by attained age $a$, i.e., baseline risk for lung cancer (in absence of exposure) at age $a$ conditional on survival up to age $a$


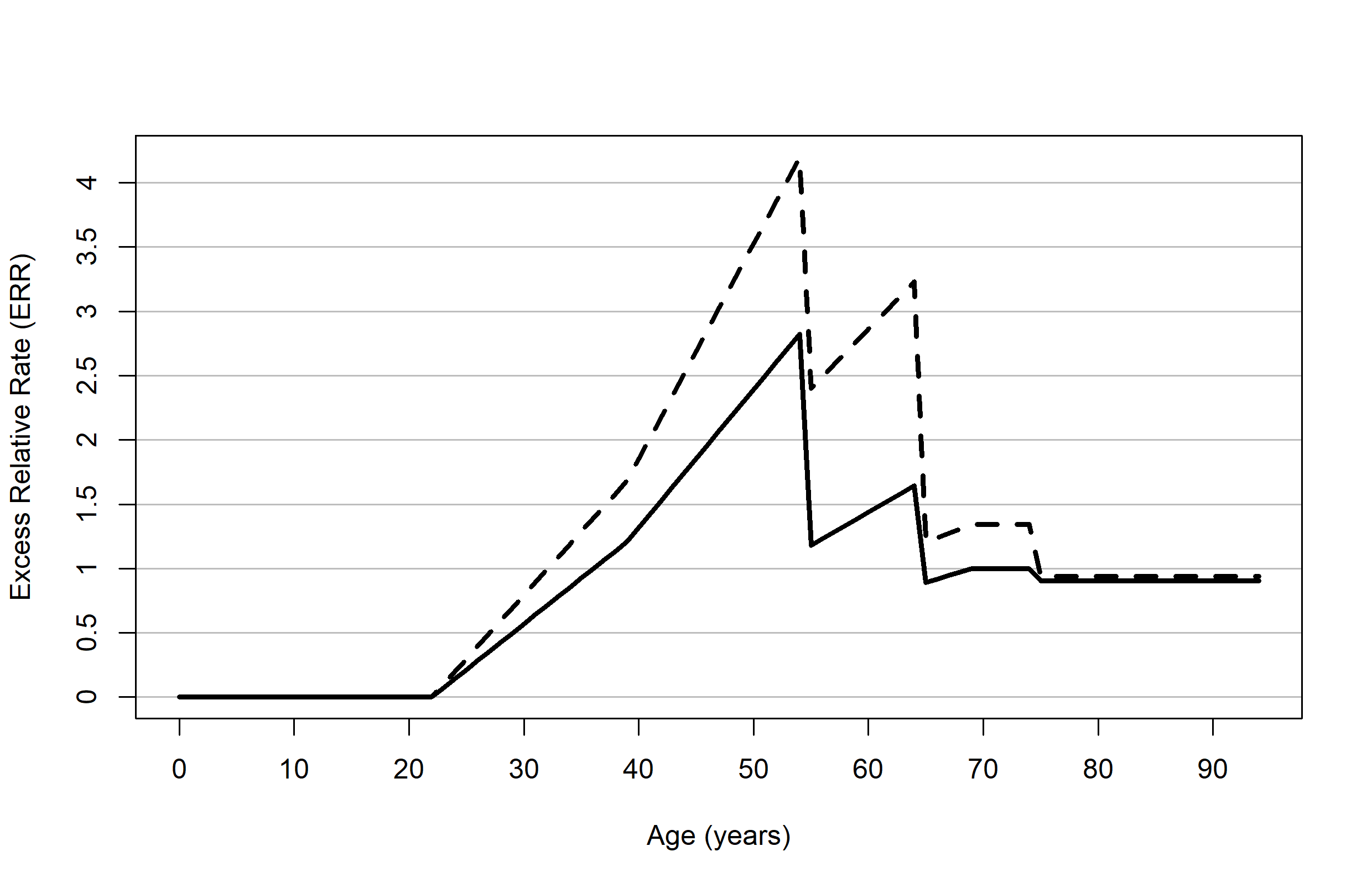


**
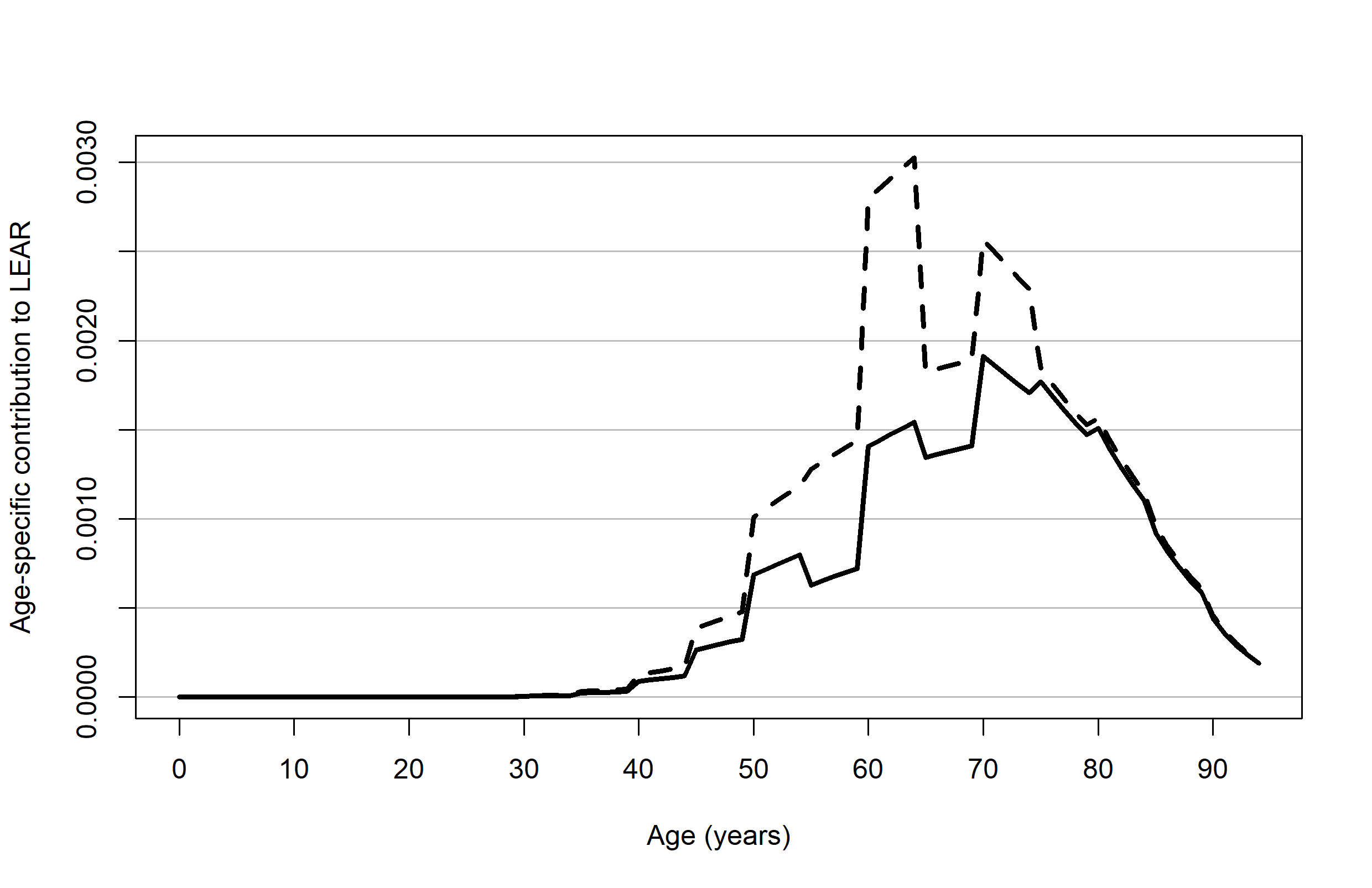
**

**Suppl. Figure 2**: LEAR components by attained age (**Upper part**: $\text{ERR}(a)$, **Bottom part**: age-specific contribution to LEAR, $r_{0}\left( a \right)\text{ERR}(a)\tilde{S}(a)$) predicted in the full PUMA cohort (Kelly-Reif et al. 2023, solid line) and the PUMA 1960+ sub-cohort (Richardson et al. 2022, dashed line) by attained age for the exposure scenario of 2 working level months (WLM) per year from age 18 to 64 up to age <95 years, assuming a 5-year lag for the model with effect modifiers attained age, age at exposure and exposure rate, and using baseline mortality rates derived from the ICRP mixed Euro-American-Asian population (ICRP 2007)
